# Case Report: An exceptional localisation in the thigh of a giant primary subcutaneous hydatid cyst.

**DOI:** 10.12688/f1000research.157733.1

**Published:** 2024-10-21

**Authors:** Aymen Fekih, Jacem Saadana, Firas Chaouch, Firas Boughattas, Ikram Haddada

**Affiliations:** 1 Fattouma Bourguiba University Hospital of Monastir, Orthopaedic Department, University of Monastir, Monastir, Tunisia; 2 Taher Sfar University Hospital, Physical Medicine Department, University of Monastir,, Mahdia, Tunisia

**Keywords:** Hydatid cyst, Subcutaneous, Primary, Thigh

## Abstract

Hydatid disease is a zoonotic disease caused by the larval stage of Echinococcus granulosus and is found globally. Although the liver and lungs are the most prevalent sites of involvement, sub-cutaneous hydatid cysts are rare especially in the primary occurrence. Sub-cutaneous hydatid cysts usually are noticed as slow growing, painless, and mobile masses beneath the overlying skin which is usually normal and are generally found on the trunk and proximal extremities. To the best of our knowledge, very few cases of primary subcutaneous hydatid cysts of the thigh have been documented. We present a case of a 30-year-old woman from a rural setting, who has a large, painless swelling on her thigh. Imaging studies confirmed the presence of a primary subcutaneous hydatid cyst, which is an atypical presentation of the disease. The entire lesion was excised surgically without any evidence of recurrence for a long period postoperatively. This case illustrates the difficulty in establishing a diagnosis and the importance of early surgical management in these cases.

## Introduction

Alveolar echinococcosis is a zoonosis which is prevalent in all geographical regions of the earth, due to the infection which occurs in the development stages in humans or animals of Echinococcus granulosus. ^1^ Most commonly, the liver and lungs are the ailing organs, and the brain follows. Such parasitic infection of primary cutaneous site of invasion is exceptionally rare and scantily available in literature. ^2^

This study reports the case of a young woman presenting with a giant subcutaneous hydatid cyst primarily localized to the thigh, which ultimately required surgical management.

## Case report

A 30-year-old female from an endemic area came with complaint of painless subcutaneous mass on right thigh.

**Figure 1.**
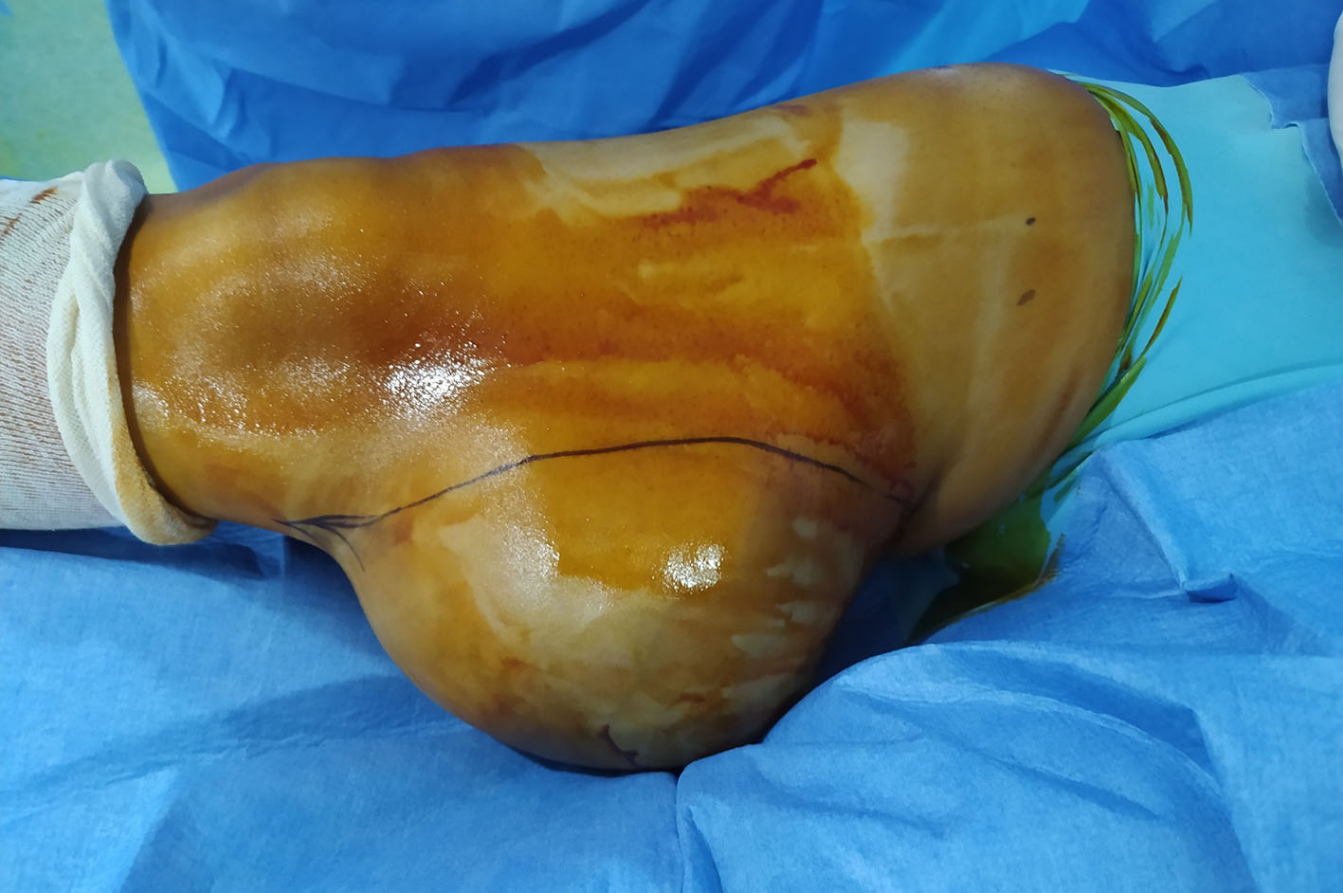
Clinical appearance of giant tumor of the inner thigh with skin ulceration.

Examination findings established a firm, fixed, 20 cm diameter mass with a superficial ulcer in the overlying skin ( Figure 1). This mass was discovered nine months ago and has gradually increased in size, becoming genate to walking.

**Figure 2.**
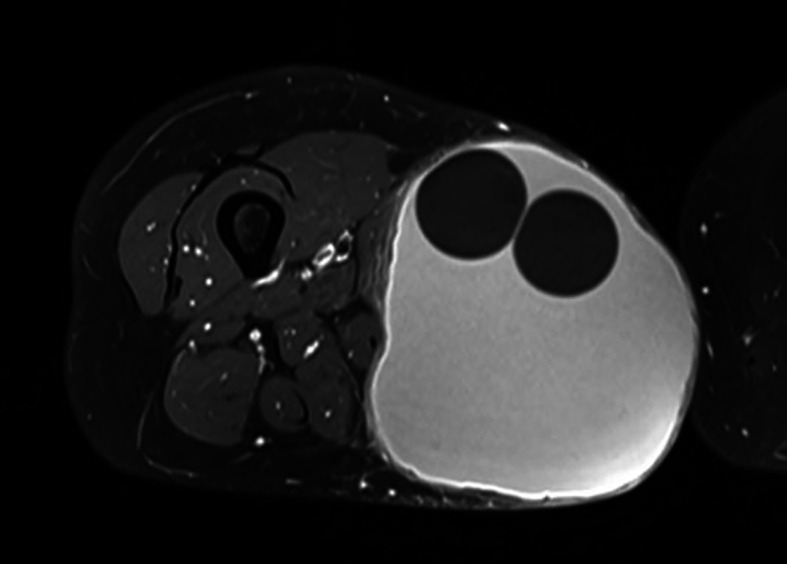
MRI appearance of the cyst showing daughter cysts in its center and a subtle peripheral enhancement after gadolinium injection.

A magnetic resonance imaging (MRI) showed a singular large subcutaneous lesion measuring 166×120×123 mm, which possessed a daughter cyst in the middle and appeared bright in T2 weighted sequence and had peripheral enhancement after the administration of gadolinium, indicating a hydatid cyst ( Figure 2).

Since serology returned negative and there were no other abnormalities on assessment of other sites, primary subcutaneous hydatid cyst was diagnosed.

**Figure 3.**
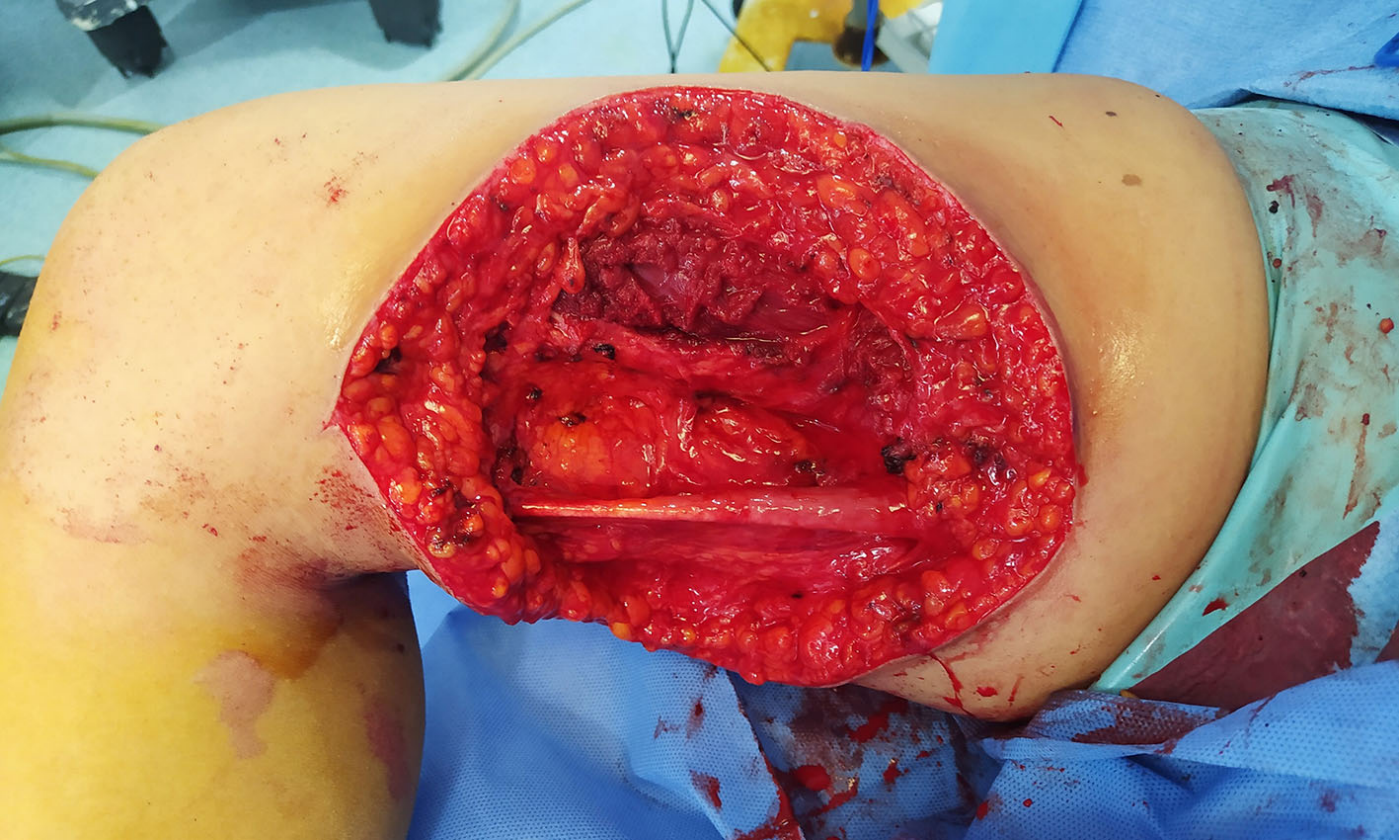
Intraoperative view showing complete resection of the lesion sparing the adductor muscles.

**Figure 4.**
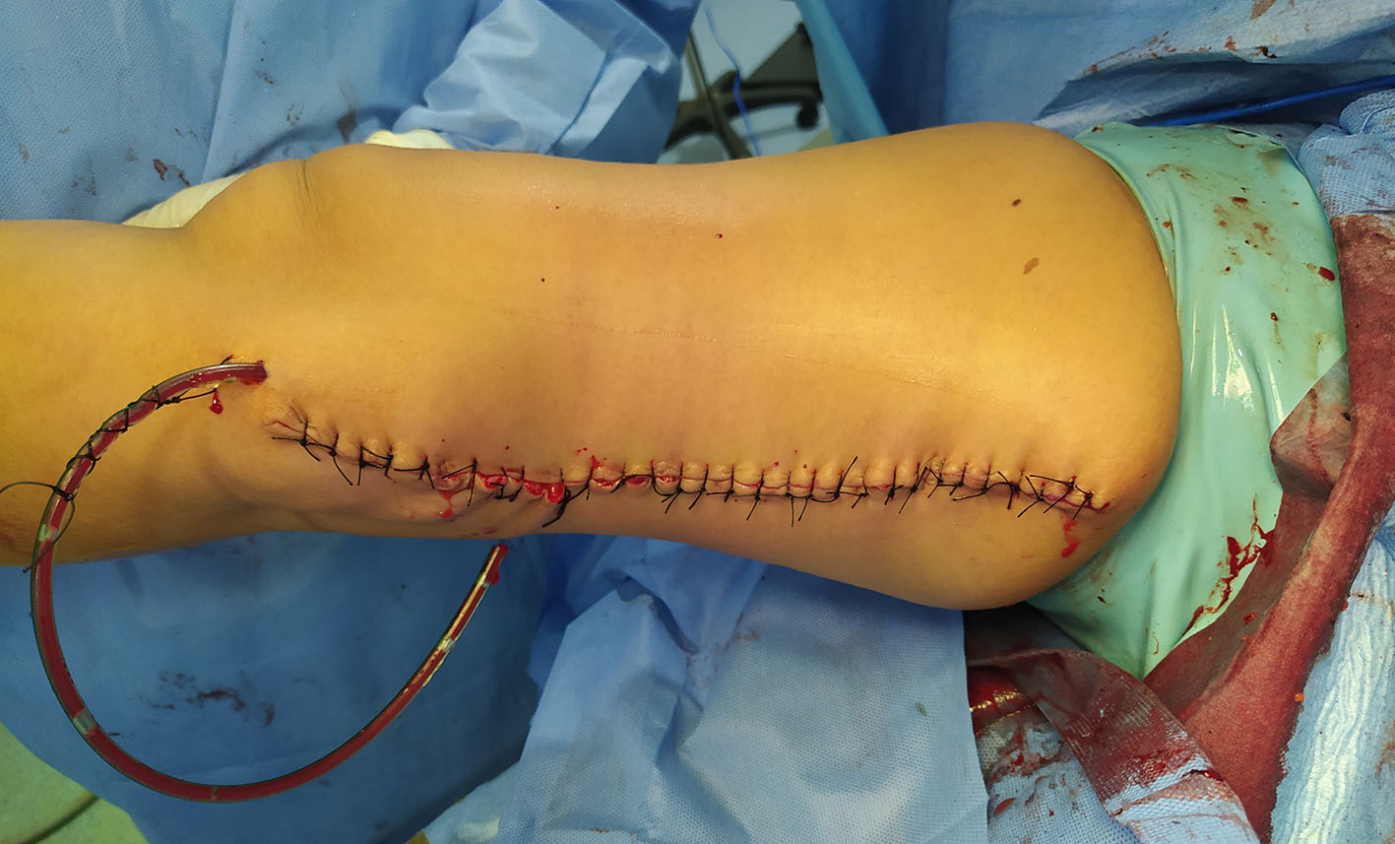
Closure of the surgical wound without the need for a covering procedure.

Surgical management involved complete excision of the lesion with sufficient margins, which preserved the adductor muscle complexes ( Figure 3). Despite the apposition of a tension-free primary closure, a skin defect created by the excision of the cyst was also present ( Figure 4).

The diagnosis of hydatid cyst was confirmed by histological examination. Anthelmintic treatment was not undertaken, as the serological results were negative.

After the procedure, the recovery was uncomplicated. The patient has been under observation for two years with no evidence of either local or distal recurrence.

## Discussion

Hydatid disease, a parasitic infection caused by tapeworms of the genus Echinococcus is considered a serious health issue in such areas. This endemic disease caused is transmissed through ingestion of food or water likely to contain the tapeworm eggs. ^3^

Most of the hydatid cysts do develop in the liver and lungs; however, primary isolated subcutaneous localization is infrequent and accounts for rates of about 2%. ^4^

The cause of primary subcutaneous hydatid cysts formation has not been established however, several explanations have been suggested: such as contamination of skin lesions, or eggs which have bypassed the liver and lungs, and have settled in the subcutis. ^5^

The typical course of subcutaneous hydatid cysts is characterized by a slowly enlarging, painless and mobile mass under intact overlying skin. ^6^

It should be noted that complications like infection or cyst rupture, while uncommon, can happen, and therefore treatment should be sought immediately. ^7^

In contrast to visceral hydatid cysts, cysts located in the subcutaneous region scarcely enlarge inwards resulting in minimal pressure effects on adjacent parts. ^8^

Imaging modalities, particularly ultrasound, play a significant role in the assessment of subcutaneous hydatid cysts. Indeed, the structure of cysts can show thick walls with daughter cysts inside, calcifications and a special germinal layer. ^6^

MRI is usually the preferred imaging modality for assessment of the soft tissue tumors located outside the bone, while ultrasound and computed tomography (CT) scan are considered as alternative modalities. ^9^

As for the subcutaneous hydatid cysts, the imaging technique that is mostly preferred is the ultrasonography. In this regard, it is worth noting that according to the literature review carried out by Kayaalp, ultrasonography, especially conducted by seasoned radiologists, enables the clear capture of such images and gives an accurate diagnosis to the patients. Cyst proximity to other organs can be evaluated utilizing CT scan and MRI. ^6^

The therapy of choice for the subcutaneous hydatid cysts usually consists of medical treatment and surgery.

While considering the treatment of such cysts, radical surgical resection of the cyst is indicated, however significant precautions are taken to avoid rupturing the cyst or disseminating the parasite within the surrounding soft tissues. ^8^ This is usually done by placing the operating field under protection with hypertonic saline soaked packs. Considering the dimension of subcutaneous hydatid cysts, a considerable amount of skin can be lost during the surgical excision often requiring skin flaps. ^5^

However still, in the case of our patient, it was possible to perform primary closure even though the cyst was large.

Most patients are prescribed albendazole as an adjunctive therapy for helminthic infestations that aims at decreasing the number of live cysts present as well as the chances of new infestations occurring. ^6^

There were few complications after the operative removal of subcutaneous hydatid cysts and the chances of recurrence after complete surgical resection were very low. ^10^ However, regular visits are important to monitor for any emergence of new growths.

In the context of the detection of a subcutaneous hydatid cyst, one should also look for the presence of other such cystic lesions in the body in the most efficient way. ^6^

## Conclusion

Primary subcutaneous hydatid cysts may be present in layers or tissue masses that may resemble soft tissue tumors; however, this is more dominant in areas where this disease is prevalent. Other visceral involvement should be looked for very carefully. The only way to avoid recurrence is to resect the lesion completely without any rupture.

## Consent

Written informed consent for publication of their clinical details and/or clinical images was obtained from the patient.

## Author contributions

**Aymen Fekih:** Project Administration, Supervision, Validation, Visualization, Writing – Original Draft Preparation, Writing – Review & Editing

**Jacem Saadana:** Methodology, Project Administration, Resources, Supervision, Validation, Visualization

**Firas Chaouch:** Conceptualization, Supervision, Validation, Visualization

**Firas Boughattas:** Conceptualization, Visualization

**Ikram Haddada:** Investigation, Resources, Validation, Visualization

## Data Availability

All data underlying the results are available as part of the article and no additional source data are required.
