# Supplementary material for: Case Report: An exceptional localisation in the thigh of a giant primary subcutaneous hydatid cyst
Source: F1000Res. 2024 Oct 21;13:1259. [Version 1] doi: 10.12688/f1000research.157733.1 (PMC11803403; doi:10.12688/f1000research.157733.1)
Supplement: Peer review history

# Reviewer report: Reviewer response for version 1

**DOI:** 10.5256/f1000research.173227.r353492

**Published:** 2024-12-30

**Authors:** Alin Mihetiu, Bogdan Vintila

**Affiliations:** 1 Faculty of Medicine, County Clinical Emergency Hospital of Sibiu, Lucian Blaga University of Sibiu, Sibiu, Romania

This article provides a well-structured presentation of this rare location of hydatid disease.

 The introduction makes a brief overview of the hydatid disease and its unusual locations, but it could also review other locations than those presented (pancreas, spleen, eyes, etc.).

 The case report part is well organized with good information and a smooth thread of presentation both in terms of the diagnostic stages and those of operative technique and postoperative evolution.

 The discussion chapter is well structured and succinctly touches on all the elements that build the approach to such a case.

Are enough details provided of any physical examination and diagnostic tests, treatment given and outcomes?

Yes

Is the case presented with sufficient detail to be useful for other practitioners?

Yes

Is sufficient discussion included of the importance of the findings and their relevance to future understanding of disease processes, diagnosis or treatment?

Yes

Is the background of the case’s history and progression described in sufficient detail?

Yes

Reviewer Expertise:

General Surgery, Hepatobiliary and Pancreatic Surgery, Laparoscopic Surgery, Oncological Surgery

We confirm that we have read this submission and believe that we have an appropriate level of expertise to confirm that it is of an acceptable scientific standard.

# Reviewer report: Reviewer response for version 1

**DOI:** 10.5256/f1000research.173227.r340747

**Published:** 2024-12-10

**Authors:** Binod Paudel

**Affiliations:** 1 Pokhara Academy of Health Sciences, Pokhara, Nepal

It feels interesting while going through this case report of subcutaneous hydatid cyst. Background , Case history, Examination findings and discussion section are clearly explained. Image section are congruent with the text section and also, references are fine.  I feel confident that this case report will help in teaching learning process.

Are enough details provided of any physical examination and diagnostic tests, treatment given and outcomes?

Yes

Is the case presented with sufficient detail to be useful for other practitioners?

Yes

Is sufficient discussion included of the importance of the findings and their relevance to future understanding of disease processes, diagnosis or treatment?

Yes

Is the background of the case’s history and progression described in sufficient detail?

Yes

Reviewer Expertise:

Radio-medicine

I confirm that I have read this submission and believe that I have an appropriate level of expertise to confirm that it is of an acceptable scientific standard.
